# The salutogenesis perspective of Health Literacy

**DOI:** 10.1093/eurpub/ckac129.515

**Published:** 2022-10-25

**Authors:** L Saboga-Nunes, M Medeiros

**Affiliations:** 1 Institute of Sociology, University of Education, Freiburg, Germany; 2 ISAMB, Institute of Environmental Health, Lisbon, Portugal

## Abstract

**Introduction:**

Health Literacy (HL) is the third pillar of Health Promotion and contributes to the sustainability agenda set earlier in Rio de Janeiro and latter in Shangai (e.g. WHO Shanghai Declaration, 2016). Nevertheless HL per se lacks consistency and structural modus faciendi implementation and practice. Therefore it is proposed that a discussion be developed based in the salutogenesis embodiment of health literacy from the perspective of children's Sense of Coherence.

**Methods:**

A population cross-sectional study included (n = 725) children enrolled in the school year 2018/2019 of four schools in Santarem and Lisbon, Portugal. Indicators of anthropometric data, Health literacy (HL), water intake (WI) and nutrition status (NS) and their Sense of Coherence (SoC) levels (using the HLS-EU-PT questionnaire) were collected with the CrAdLiSa online self-administered questionnaire.

**Results:**

Preliminary results show that the instrument to measure HL (HLS-EU-PT) show satisfactory internal consistency (Cronbach's alpha coefficient 0,94) and association with SoC (Cronbach's alpha coefficient 0,89). The higher is HL levels the higher results are found in the Comprehensibility component of the SoC. The older the age, the amount of WI perception is adequate (p = 0.03); male children (p = 0,02) and children that attend schools in urban area (p = 0,01) drink more water; and have a higher SoC. Also the older the age, the lower the levels of HL (p = 0.000); children with higher levels of HL have greater WI (p = 0.01) as they have a broader perception of the adequate amount of water needed to be healthy.

**Conclusions:**

These results of CrAdLiSa study explore further on the theoretical perspectives influencing HL. They also showed the usability of the SoC and the HLS-EU-PT questionnaires adapted for children to assess their SoC and HL levels.

